# A War Victim

**Published:** 1871-03

**Authors:** 


### A WAR VICTIM.

We regret to hear of the death, at Versailles, of Dr. Brewster, an American dentist, who long practiced in Paris, and attended many of the crowned heads of Europe. A few years ago he retired in favor of Dr. Evans, and took up his residence at Versailles, where, notwithstanding the American flag floating over his house, a number of Prussian officers were quartered upon him. Dr. Brewster died on Thursday, Dec. 15th, in a fit, doubtless brought on by the excitement and trouble in which he has been living for the last few months.—*Lancet.*

